# Analysis of Impact of Rain Conditions on ADAS

**DOI:** 10.3390/s20236720

**Published:** 2020-11-24

**Authors:** Chang-Gyun Roh, Jisoo Kim, I-Jeong Im

**Affiliations:** Smart Mobility Research Center, Department of Future Technology and Convergence Research, Korea Institute of Civil Engineering and Building Technology, Gyeonggi-do 10223, Korea; rohcg@kict.re.kr (C.-G.R.); js.kim0331@kict.re.kr (J.K.)

**Keywords:** lane departure warning system, advanced driver assistance systems, vehicle-based driving experiment, weather condition, rainfall

## Abstract

Various technologies are being developed to support safe driving. Among them, ADAS, including LDWS, is becoming increasingly common. This driver assistance system aims to create a safe road environment while compensating for the driver’s carelessness. The driver is affected by external environmental factors such as rainfall, snowfall, and bad weather conditions. ADAS is designed to recognize the surrounding situation and enable safe driving by using sensors, but it does not operate normally in bad weather conditions. In this study, we quantitatively measured the effect of bad weather conditions on the actual ADAS function. Additionally, we conducted a vehicle-based driving experiment to suggest an improvement plan for safer driving. In the driving experiment, when the vehicle driving speed was changed in four stages of rainfall, it was confirmed that it affected the View Range value, where the primary variable is the visibility of ADAS. As a result of the analysis, we demonstrated that when the rainfall exceeded a precipitation of 20 mm, the ADAS sensor did not operate, regardless of the vehicle speed. This means that a problem affecting safe driving may occur due to functionality in bad weather situations in which the driver requires ADAS assistance. Therefore, it is necessary to develop a technology that can maintain the minimum ADAS functionality under rainfall conditions and other bad weather conditions.

## 1. Introduction

The lane departure warning system (LDWS) is one of the mechanisms of advanced driver assistance systems (ADAS) that increase driving safety by providing a warning to the driver when a vehicle driving on the road leaves the driving lane. Most current traffic accidents are caused by the driver’s carelessness. Therefore, the various technologies constituting ADAS can be said to be the main technologies for safe driving and securing safety on the road [1,2,3]. The system is designed to minimize the accidents caused by driver’s negligence, such as drowsy driving and unexpected situations while driving. Since 2009, the National Highway Traffic Safety Administration (NHTSA) has begun a study to make it mandatory to introduce a lane departure warning system and a frontal collision warning system [4,5]. In Korea, LDWS, one of the ADAS technologies, has been installed in some luxury vehicles to support safe driving. However, in recent years, as large-scale traffic accidents have occurred due to the carelessness of the drivers of trucks and heavy transport vehicles, LDWS installation has been mandatory for buses and trucks since July 2017 to prevent accidents [6].

However, general driver carelessness may be caused by driver-related factors, but various external factors also affect the driver’s driving, and some external factors may also cause driver carelessness [7,8]. It is not simply the driver’s carelessness that causes traffic accidents, and meaning that a complex of factors can result in driver carelessness, and will likely lead to accidents. Self-driving cars use sensors such as LIDAR, Radar, Camera, and Mobileye to provide ADAS functions, including LDWS. Since the vehicle drives itself and is not driven by ordinary drivers, the sensor function is the core of self-driving technology. As the development of self-driving cars has recently become more active, the safety of self-driving is being discussed. Opinions are largely divided on whether the installed sensors are able to maintain sensor function even under the bad weather conditions mentioned above in order to enable safe driving [9]. Hadi, M. et al. (2007) [10] analyzed the effect of environmental factors on LDWS image recognition. As a result of this study, it was shown that the visibility was poor under bad weather conditions with heavy rainfall. Additionally, in other research and analysis results on the rainfall environment’s influence on the LIDAR sensor used in ADAS, the sensor showed limited rainfall recognition. This means that empirical studies using complex sensors and considering regional factors are necessary [11,12]. Similar to rain conditions, some research has analyzed the effects of fog conditions on ADAS. To solve the difficulty of securing sensor visibility in fog conditions, Jeong, K. M. et al. (2018) developed an algorithm for fog detection and fog removal. Additionally, they suggested a method for maintaining the ADAS function by using image data in fog [13]. Tumas P. et al. (2020) used a detector based on a Deep Neural Network (DNN) to enhance the awareness of pedestrians of the ADAS system under bad weather conditions using the Controller Area Network (CAN) data collected through ADAS. They tested the ability to recognize pedestrians under situations with different weather conditions. The experiment confirmed that the temperature and the car’s speed act as significant factors, and that additional experiments in various weather environments are necessary [14]. Some research has used image analysis to confirm the effect of rainfall conditions and illumination on ADAS vision. This research supplemented ADAS functions such as road recognition and intelligent vehicle control with image analysis in the driving environment considering various weather and lighting conditions [15,16]. Hasirlioglu, S. and Riener, A. (2017) analyzed the effect of adverse weather conditions on primary sensors (Camera, LiDAR, radar) through simulation under adverse weather conditions such as rain and fog. As a result of the study, it was confirmed that cameras and LiDAR sensors, which are essential for ADAS function, are affected by bad weather, suggesting that it is necessary to compensate for sensor performance, which deteriorates according to the driving environment [17]. Hadj-bachir, M. and De Souza, P. (2019) simulated LiDAR in severe weather conditions. Their results confirmed LiDAR’s decreasing intensity in severe weather conditions and the effect of bad weather conditions on LiDAR visibility. Additionally, they proposed an improvement plan for LiDAR through a virtual sensor [18]. The Disengagement Reports of the California Department of Motor Vehicle (DMV) [19] provide data on accidents resulting from the release of self-driving systems. Several studies using the Disengagement Reports data have analyzed the factors affecting the release of self-driving systems. They concluded that adverse weather conditions, such as rainfall and snowfall, affect accidents [20,21,22,23]. Additionally, based on the studies, another study that derives and analyzes factors affecting self-driving cars’ driving safety also confirmed that bad weather conditions need to be studied first to ensure driving safety [24].

Recognizing lanes is a basic function of LDWS and is a major technology that can increase driving safety. It is important to accurately recognizing the surroundings while driving, but it is more important to maintain a consistent driving performance. In other words, consistent driving performance must be maintained in various environments, and even in bad weather conditions such as rainfall or snowfall, and ADAS should not interfere with the function or cause problems with vehicle driving. However, the performance of LDWS, which is legally mandated for the safety of drivers and traffic participants, is being evaluated in ideal situations such as daytime on a sunny day, dry weather, and lanes with guaranteed visibility. In ideal traffic conditions, there are not many situations that hinder safe driving. However, the risk of accidents is higher in situations where there are external factors that actually affect driving. Therefore, in terms of LDWS supporting safe operation, LDWS performance evaluation methods should be introduced for situations where various problems could arise, rather than carrying out performance assessments based on ideal circumstances.

Therefore, in this study, we intend to conduct an actual vehicle-based driving experiment to evaluate the performance of LDWS under rainfall conditions. First, we reviewed the existing LDWS performance evaluation methodology and the current performance evaluation methodology’s limitations. Second, to confirm the ability of LDWS to implement performance in a non-ideal driving environment an experimental environment capable of reproducing the rainfall environment was established to confirm LDWS performance with respect to rainfall. Finally, based on the previously derived results, we derive the problem of LDWS performance and performance evaluation methodology, and we propose a revised method.

## 2. Review of LDWS Evaluation Standard

### 2.1. International Standard for LDWS

As ADAS technologies have progressed rapidly, international and national standards have been developed for the safety and testing of LDWS. ISO standards provide specific requirements for LDWS and standards for testing. The specific requirements present specifications for the road environment and system environment for correct LDWS implementation. Additionally, they standardize and offer specific environmental conditions, including road geometry and weather, in the test method [25]. US SAE presents a test method for LDWS for OEM and aftermarket. Like the ISO standards, it gives details on the driving environment, such as specific road geometry and weather [26]. Additionally, the United States’ NHTSA proposes specific test methods, similar to those of ISO and SAE, and suggests specific test methods so that LDWS-related stakeholders can implement safe systems [27]. Table 1 shows a summary of the specification of each standard.

### 2.2. Domestic Standards (South Korea)

There are three standards related to LDWS in Korea. The standards defining the device’s performance test method are ‘Performance Test of Lane Departure Warning Device for Passenger Cars—Definition and Test Method of Road and Environmental Conditions’ (KS R 1172) and the Regulations for Vehicle Safety Assessment Test (the Ministry of Land, Infrastructure, and Transport (MOLIT), No. 2018-70). ‘Rules on the performance and standards of automobiles and automobile parts (MOLIT, No. 534; partially amended on 11 July 2018)’ is a standard defining equipment performance standards.

The test vehicle regulations, test speed, road, environmental conditions, regulations for lane recognition, and performance standards are all different, as shown in Table 2. The criteria, except for KS R 1172, are all ideal conditions (including clear day, dry road) and prescribe testing under the specific conditions of painting lanes.

MOLIT established the ‘Enforcement Regulations of the Traffic Safety Act’ in 2017 as performance standards. This mandated the installation of LDWS on buses and freight vehicles, and suggested following ‘Vehicle Safety Evaluation Test’ and ‘Performance and Standards of Automobiles and Auto Parts’. Accordingly, this means that ADAS (LDWS) equipment that can be attached and used under the ‘Enforcement Regulations of the Traffic Safety Act’ does not have to operate the warning function in bad weather.

### 2.3. Summary and Implications

As a result of reviewing the existing standards, all standards except for “KS R 1172”, one of Korea’s performance test method standards, require performance tests of equipment to be carried out based on the results determined for ideal conditions. Thus, this means that drivers may not receive assistance in situations that require assistance for safe driving in bad weather conditions. Regardless of the type of information collected and the reliability of the information, we cannot verify detailed data because the performance evaluation results were based only on the presence or absence of a lane departure warning being transmitted to the driver.

No evaluation criterion suggests the type of data detected by the LDWS equipment, their accuracy, or the reliability criteria for the data. Therefore, if the lane departure alarm provided by the ADAS (LDWS) equipment to the driver satisfies only the performance evaluation criteria range, this means that it can be judged to be suitable equipment and supplied and used regardless of the reliability and accuracy of the collected data. Therefore, in this study, we confirmed the function of LDWS in a rainfall environment by analyzing collected data under rainfall conditions.

## 3. Lane Departure Warning System Performance Test Methodology

### 3.1. Performance Test Equipment

**Figure 1:**
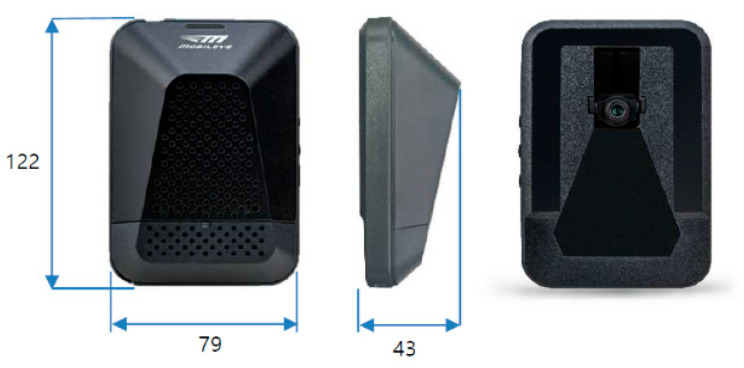
Mobileye 630 camera unit.

In this study, ‘Mobileye 630’ by Mobileye was used as LDWS performance test equipment. This equipment is the most widely used LDWS equipment in the world, and is known to provide the most reliable information. We collected detailed information through the Mobileye 630 and CAN communication. Photos of the equipment and the information collected are presented in Figure 1 and Table 3.

### 3.2. Properties and Characteristics of Collected Data

#### 3.2.1. Properties of Collected Data

From the Mobileye 630 detection data collected through the lane departure detection device performance test equipment, on the basis of an analysis of the lane-related information and data affected by bad weather (rainfall), a total of four data types were derived. The data types and attributes (definitions) affected by bad weather (rainfall) in Mobileye’s data protocol were as follows. Among the four main lane-related variables derived, View Range was the variable related to visibility, and the other three variables were information related to the accuracy of the lane information. This includes the visibility of the driver’s side, because the View Range information describes the distance from the current position of the vehicle to the most distant lane recognized by the ADAS (LDWS).

The detailed characteristics of the main variables related to the four lanes are as follows. As previously suggested, View Range refers to the distance to the farthest object that ADAS (LDWS) can recognize. Lane Type and Width left (right) Marking are accurate attribute information of a nearby lane, and Quality is the determination of whether the information was sufficiently obtained when the ADAS (LDWS) was determining lane information. That is, among the information collected for ADAS (LDWS), only the View Range is a variable that can determine the influence of visibility under bad weather conditions. Lane Type, Width left (right) marking, and Quality are other information accuracy variables that can determine whether the driver is informed by accurately recognizing the properties of the lane located in the distance. The detailed property information for each variable is as follows:

1. View Range
  - Distance from the ADAS (LDWS) attached to the vehicle to the farthest obtained lane, the range of ADAS (LDWS) visibility in each situation, and time point
  - Value within the range of 0 to 127.996 m (actual range on the basis of test driving: 0 to 80)
2. Lane Type
  - Classified into a total of six types
  - 0: dashed; 1: solid; 2: undecided; 3: road edge; 4: double land mark (including dashed on one side); 5: Botts’ dots; 6: invalid
3. Width left (right) marking
  - Thickness of the lane on the left (right) side of the vehicle (in meter)
4. Quality
  - Expresses the quality of lane information in a range of 0 to 3
  - 0, 1: low quality, not give an LDWS in that situation; 2, 3: High quality
  - It is possible to collect lane information even in the situation of Quality 0 or 1, and LDWS alarm is provided using the collected information

#### 3.2.2. Data Characteristics

**Figure 2:**
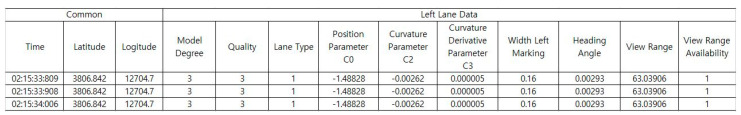
ADAS Data Format.

Data are collected between 6 and 11 times per second, with an average of 9.4 times, through CAN communication. As shown in Figure 2, each dataset is collected and stored separately for the left and right lanes, and a total of 10 fields are collected for each lane, as shown in the following figure. That is, all data from the model degree that recognizes the lane to the reliability of the View Range are recognized as separate lanes.

**Figure 3:**
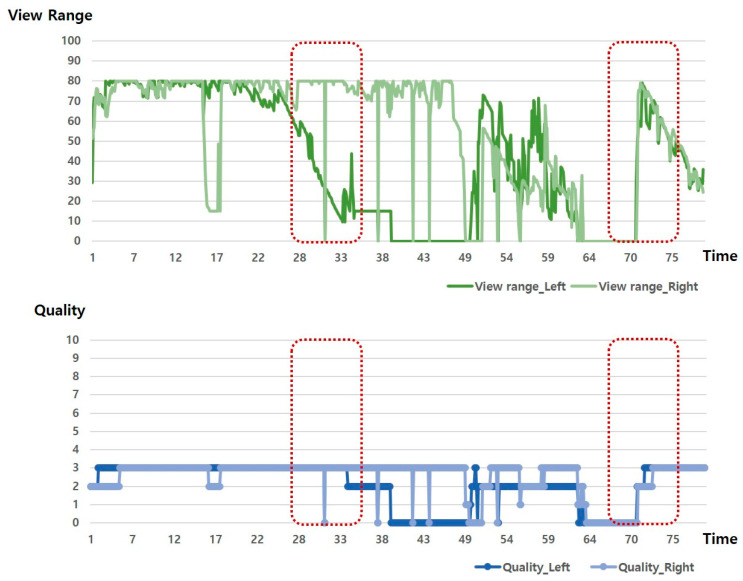
Relationship between View Range and quality.

Figure 3 is a schematic diagram that shows the relationship between View Range and Quality. Although the pattern changes for View Range and Quality value are similar, as shown in the section indicated by the red dotted line, the quality is maintained or increased even when the View Range decreases. In other words, View Range and Data Quality are in a mutually independent relationship.

**Figure 4:**
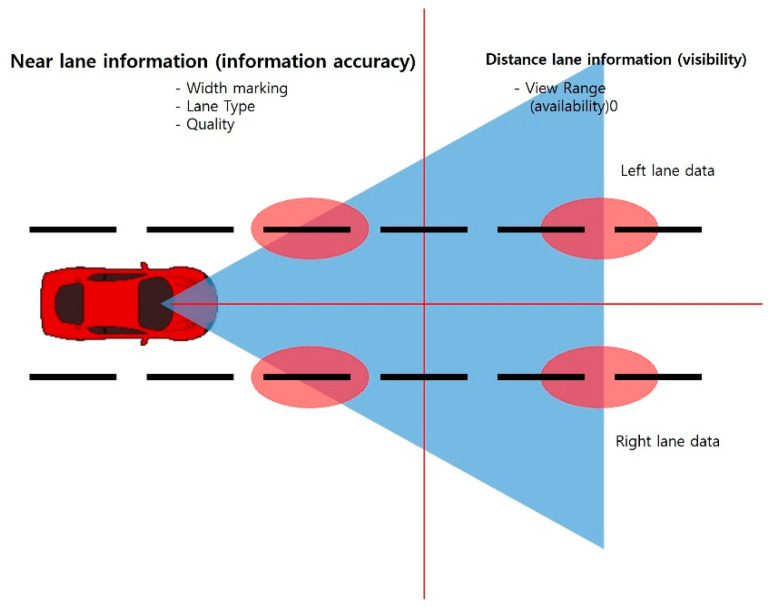
ADAS data characteristics.

Due to the characteristics of ADAS (LDWS), information related to View Range is information on a long-distance lane, and Lane Tape and Width left (right) marking are information on a side lane of the vehicle. Figure 4 presents a schematic of this. ADAS (LDWS) obtains information on distant lanes separated by more than a certain distance ahead and collects continuous lane information. However, in order to determine whether the front wheel of the vehicle has deviated from the lane and give an alarm, the lane information on the side of the vehicle at the same time is processed together. Therefore, even with data on the same row collected at the same time, the lane information of the corresponding row can be said to be lane information at different locations. In this study, data analysis was conducted considering this.

### 3.3. Experiment Methodology

**Figure 5:**
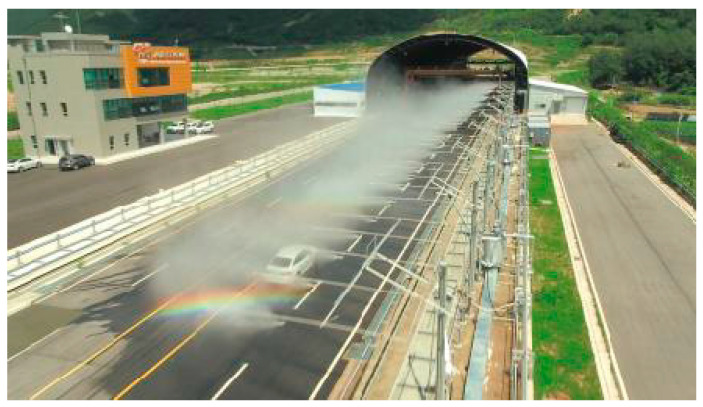
Reproduction of rainfall environment and view of experimental environment.

To confirm the change in ADAS performance due to the rainfall, the change in the View Range collected by ADAS was analyzed while changing the rainfall to 0 mm, 10 mm, 20 mm, and 30 mm. The vehicle speed was tested based at three speeds: 48 km/h (In case of middle rain: 20% reduction in speed limit), 30 km/h (In case of heavy rain: 50% reduction in speed limit), and 60 km/h (General speed limit in Urban area), considering the reduction in rainfall. The experiment was conducted at the Center for Road Weather Proving Ground in Yeoncheon, Korea. Figure 5 shows the actual experimental situation, which reproduces a rainfall environment at the Center for Road Weather Proving Ground.

This test site is located in a protected military area, and the surrounding area is completely controlled. Therefore, we were able to thoroughly manage the external environment according to what was needed for the experimental setting. For the experiments, we used the LDWS of the Mobileye 630 model without modification, and the LDWS function provided by the Mobileye 630 aims to prevent the driver from changing lanes unintentionally by sounding a warning when the vehicle moves over a lane.

The vehicle used in this study was a vehicle that was specifically manufactured to collect and analyze data. However, since it was not equipped with the ADAS function, we attached the Mobileye 630 device for research to the vehicle through the Mobileye manufacturer to implement the vehicle’s ADAS function. We drove a vehicle on a fully controlled test route and collected data by controlling the amount of artificial rainfall discharged per hour through the artificial rainfall facility. Data was primarily filtered using the variables that determine the data (Lane Tape, Width left (right) marking, Quality). The change in LDWS data, according to rainfall, was analyzed using View Range data based on the filtering data.

## 4. Results of LDWS Data Characterization during Rainfall

### 4.1. ADAS (LDWS) Data Characteristic Analysis Methodology by Rainfall Intensity

**Figure 6:**
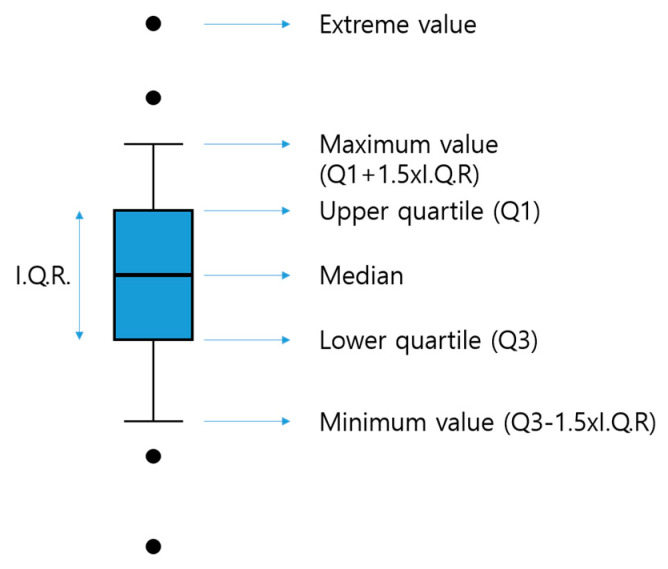
Box plot display method.

To statistically review the scatter plot of View Range Data in the same property section, the results were plotted using a box plot. The box plot (or box and whisker plot) is a method of expressing a scatter plot that makes it easy to identify the distribution and extreme values of the measured values of the data by using the maximum, minimum, median, and quadratic deviations. If the data are distributed asymmetrically, you can draw a box plot to determine the number of extreme values and whether they are asymmetrical. Therefore, it can be used as a measure of the center position and scatter of the measured value. As shown in Figure 6, a box with both ends of the first and third quartiles Q1 and Q3 is drawn using the View Range value for each section, and the median value is displayed as a horizontal line in the box. If the length of the base of the box, that is, the range Q1, Q3, is expressed as I.Q.R, a fence is drawn at ± 1.5R points and ± 3.0R points left and right from Q1 and Q3. Measurement values within the ± 1.5R point are suspect outliers, and measurement values located at the ± 3.0R point are highly suspect outliers and are marked with dot like “●” using IBM Statistics SPSS 25).

The vertical axis of the graph presented in this paper is View Range (unit: m), and the horizontal axis is ‘section characteristics rainfall (mm) vehicle speed (km/h)’. That is, ‘general rainfall_30_60′ on the horizontal axis plots the results of ‘the distribution of View Range (m) values when driving at a vehicle speed of 60 km/h when a 30 mm rainfall condition is given to the general rainfall section’.

### 4.2. ADAS (LDWS) Data Characteristic Analysis Result by Rainfall Intensity

**Figure 7:**
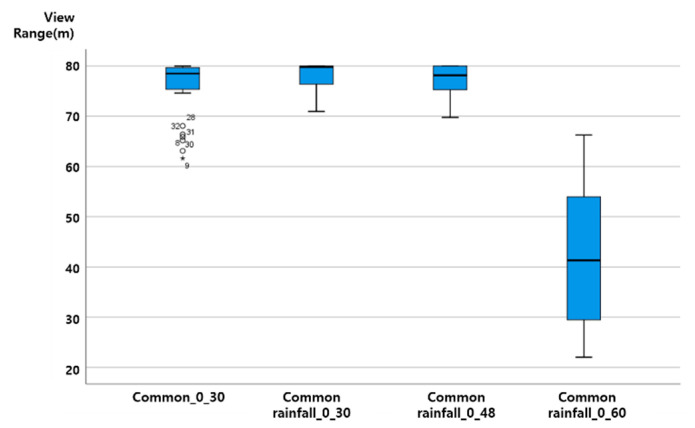
View Range change according to speed change at precipitation 0 mm.

**Figure 8:**
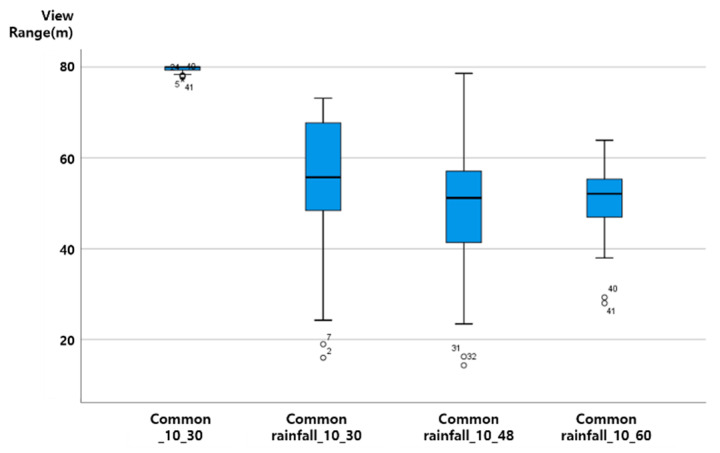
View Range change according to speed change at precipitation 10 mm.

**Figure 9:**
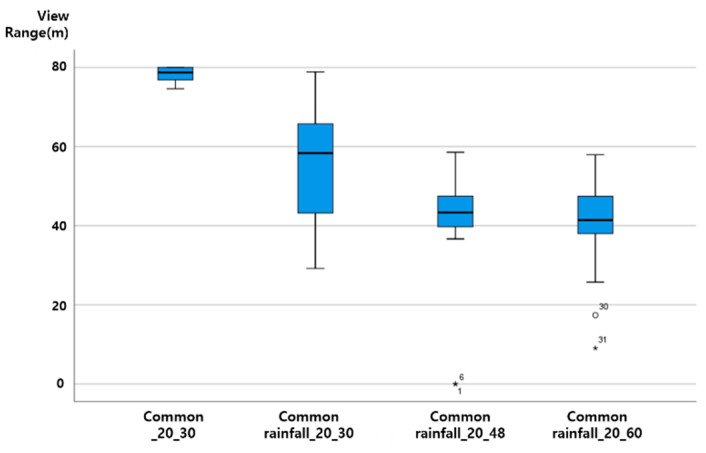
View Range change according to speed change at precipitation 20 mm.

**Figure 10:**
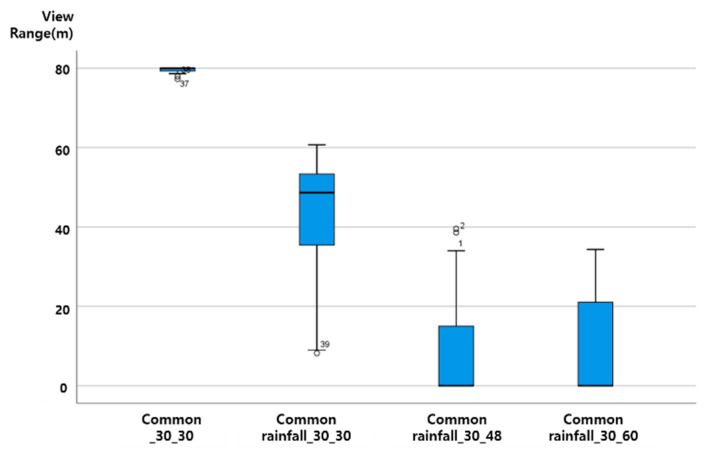
View Range change according to speed change at precipitation 30 mm.

As the vehicle speed increases in a driving environment with the same rainfall, the View Range, which is the visibility variable of ADAS (LDWS), was analyzed as shown in the following figure, as a downward-to-right distribution with decreasing View Range. Up to 20 mm precipitation, even when driving at a vehicle speed of 60 km/h, the average View Range was over 40 m. When the rainfall was 20 mm, the View Range of less than 20 m at a vehicle speed of 60 km/h was an outlier. The results from the data characteristic analysis with respect to rainfall intensity are presented in Figure 7, Figure 8, Figure 9 and Figure 10.

### 4.3. ADAS (LDWS) Data Change According to Precipitation Change

When driving at the same vehicle speed, it was analyzed that the View Range, the visibility variable, decreased in a similar pattern with 10 mm and 20 mm of rainfall. In the case of rainfall of 30 mm, the reduction in View Range was not large when driving at a vehicle speed of 30 km/h (about a 10 m decrease). However, for vehicle speeds of 48 and 60 km/h, it was confirmed through an experiment that the View Range converged to 0, and ADAS (LDWS) did not operate.

**Figure 11:**
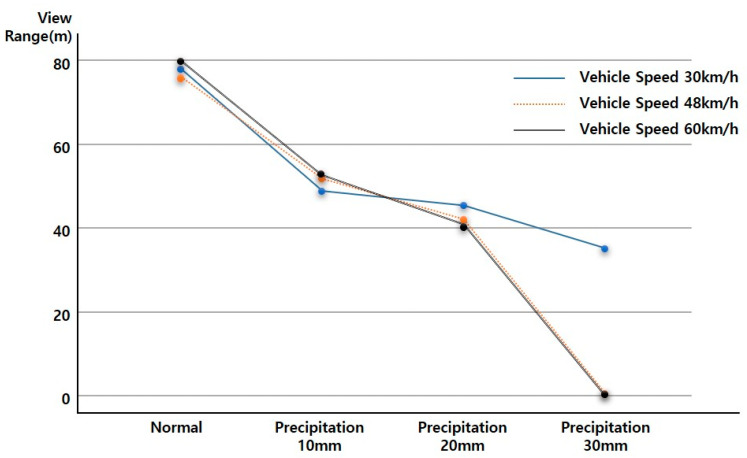
ADAS Data change according to precipitation change.

Assuming a minimum View Range of 15 m, the View Range of the transition section of ADAS (LDWS), ADAS (LDWS) can perform even when driving at a vehicle speed of 60 km/h in up to 20 mm of rainfall. However, when the rainfall is 30 mm, a different technology is required to drive the vehicle speed to below 48 km/h or to improve the visibility of the lane. The experimental results are shown in Figure 11.

### 4.4. ADAS (LDWS) Data Change According to Vehicle Speed Change

**Figure 12:**
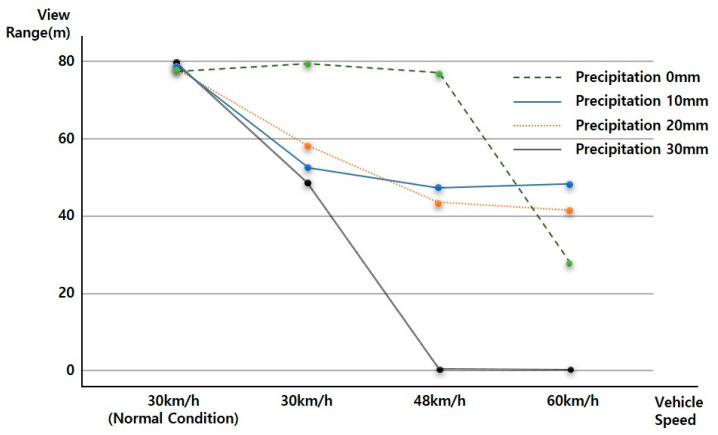
ADAS data change according to vehicle speed change.

As the vehicle speed increases in a driving environment with constant rainfall, the View Range, a downward-to-right distribution with decreasing View Range, which is the visibility variable of ADAS (LDWS), was analyzed. In addition, the results from baseline analysis are shown in Figure 12.

At up to 20 mm of precipitation, even when driving at a vehicle speed of 60 km/h, the average View Range was over 40 m. When the rainfall was 20 mm, the View Range of less than 20 m at a vehicle speed of 60 km/h was an outlier.

On the basis of the results of a more detailed review, it was confirmed that in the case of 0 mm of rainfall, the View Range was 80 m regardless of the vehicle speed, creating an ideal environment for securing visibility. However, for a vehicle speed of 60 km/h, the data needs to be supplemented through additional data analysis. As shown in the ADAS (LDWS) baseline analysis results according to rainfall change, it was determined that the View Range that ADAS (LDWS) is able to achieve at between 0 and 20 mm of rainfall is always maintained at above 40 m, which is more than 15 m. In addition, in the case of 30 mm of rainfall, it was confirmed that ADAS (LDWS) could not operate normally because the View Range became 0 m beyond a vehicle speed of 48 km/h.

However, in the case of 60 km/h, we found peculiarity that the View Range with a precipitation of 0 mm was lower than that at precipitations of 10 mm or 20 mm, because it is difficult to achieve accurate information recognition due to sunlight being recognized by the sensor in a backlight situation. This situation can happen anytime, anywhere, when driving. In particular, the View Range decreases because the sensor cannot recognize accurate information when driving at a high speed. Because of this, in cases of precipitations of 10 mm and 20 mm, we determined that the View Range increased with respect to the situation at precipitation 0 mm because the rain partially blocked the sunlight.

## 5. Discussion

This study is the result of empirical verification of ADAS performance change resulting from rainfall and changes in vehicle speed. Under rainy conditions, the driver will feel more skeptical about driving assistance systems such as ADAS. However, the current ADAS relies on an image sensor that performs the same role as the human eye. Therefore, an environment that is not able to be captured by the human eye will not exhibit performance in ADAS. We tried to confirm the limitations of LDWS and ADAS sensors by conducting a real-vehicle experiment in a rainfall environment to confirm the limitations of sensor recognition. The significance of this study is to conduct a quantitative experiment for measuring the effect of rainfall intensity on the ADAS sensor and to derive the solution for overcoming the sensor limitations by not using image data from other sensors, as in previous studies [13,14,15,16]. As a result of the empirical experiment, on the basis of the View Range reference analysis, as the rainfall increases, the ability of the ADAS sensor to identify and recognize lane information decreases rapidly. In particular, it was confirmed that when the rainfall increases, the driving speed of the vehicle also affects the View Range, such that the visibility of the surrounding environment decreases, and the safety of the vehicle driving is not secure.

Therefore, it is deemed necessary to develop a technology that can secure a View Range regardless of rainfall to ensure the safety of LDWS and ADAS technologies. Since the primary sensors are used to secure, recognize, and determine visibility for actual vehicle driving, in cases such as general drivers and self-driving cars, this will reduce accidents caused by driver carelessness. Furthermore, it is expected to provide a safe driving environment by securing the safety of self-driving cars. This study’s limitation is that we did not perform testing in other bad weather environments such as fog and snow. Therefore, future studies must consider extreme conditions (severe weather conditions) and conduct empirical research in complex environments such as fog, snowfall, and strong winds, not just rainy environments.

## Figures and Tables

**Table 1:** Summary of international standards for LDWS.

Item	ISO 17361	SAE Information Report J2808 (LDW)	NHTSA 2006-26555-0135 (LDW)
Target Vehicle	Test vehicle not esceeding 150 kg including one driver boarding and test equipment	Light-duty vehicles	Lightweight vehicle with a maximum vehicle weight class (GVWR) of 10,000
Test Speed	-	44.74 mph (72 km/h)	45 mph (72.4 km/h)
Road and environmental conditions	Flat, dry asphalt or concrete surface Radius of curvature: 250 m or 500 m Visible lane markings in good condition Temperature: 10 °C ± 30 °C.	Good weather Straight road Radius of curvature over 500 m	Good weather (Ideal conditions) Straight road
Regulation of the Lane to be Recognized	Lane in accordance with standards	-	Continuous white lines Discontinuous yellow lines Discontinuous Botts dot Raised pavement markers
Performance Standards (Criteria for passing the tests)	Warning generation test Repeatability test False alarm test	-	66% or more of the total number of times
Remark	-	Follow symbols and information provision method of ISO	-

**Table 2:** Summary of LDWS performance standards in Korea.

Classification	Performance Test Standard		Performance Standard
	KS R 1172	Vehicle Safety Evaluation Test	Performance and Standards of Automobiles and Auto Parts
Target Vehicle	No vehicle model standards Test less than 150 kg including one driver boarding and test equipment, or the maximum weight test (by agreement between consignee and deliverer, it is possible to test with the weight of 5 passengers)	Passenger cars, omnibuses and small trucks with a gross weight of 4.5 tons or less	Passenger car (excluding light-size omnibuses) Truck and special vehicles exceeding 3.5 tons of gross vehicle weight
Test Speed	100 km/h or more Over 60 km/h on highway Over 60 km/h on national highway and local roads	65 km/h ± 3 km/h	60 km/h
Road and environmental conditions	Curvature standard: ≥500 m, ≥250 m Road rank: highway, national road, local road Weather: Sunny (4 types), Rain (4 types), Snow (4 types), Fog (2 types) Others: tunnel, day/night (with street light)	Smooth and dry asphalt or concrete road surface Visible lane markings in good condition	-
Regulation of the Lane to be Recognized	-	Four types of yellow double line (center line), white dotted line and white solid line (lane), blue solid line (dedicated lane) white solid line	-
Performance Standards	-	90% or more of the total number of times	-
Remark	-	Severe weather conditions are excluded from evaluation according to the environmental conditions presented.	Warning lights on in case of bad weather such as fog or heavy rain (Lane information may not be provided)
		Domestic standards that the product must satisfy to participate in the bidding according to the Public Procurement Service announcement on the Order (Subsidy Project) due to obligatory installation of LDWS according to the revision of MOLIT.	

**Table 3:** Mobileye 630 collection data list for LDWS Operation.

Collected Data	
Common Data	Lane Data
Time Latitude Longitude	Model degree
	Quality
	Lane type
	Position parameter C0
	Curvature parameter C2
	Curvature derivative parameter C3
	Width left marking
	Heading angle
	View Range
	View Range availability
